# The Role of the Ethicist in an Interdisciplinary Transgender Health Care Team

**DOI:** 10.1089/trgh.2018.0058

**Published:** 2019-04-26

**Authors:** Hilary Mabel, Murat Altinay, Cecile A. Ferrando

**Affiliations:** ^1^ Center for Bioethics, Women's Health Institute, Cleveland Clinic, Cleveland, Ohio.; ^2^ Psychiatry and Psychology, Center for Behavioral Health, Center for LGBT Care, Cleveland Clinic, Cleveland, Ohio.; ^3^ Women's Health Institute, Obstetrics and Gynecology, Center for Urogynecology and Pelvic Reconstructive Surgery, Center for LGBT Care, Cleveland Clinic, Cleveland, Ohio.

**Keywords:** clinical ethicist, ethics, interdisciplinary team, transgender

## Abstract

Unique ethical issues arise in the provision of gender-affirming care to transgender and gender diverse people. One of the distinctive trends in transgender health care has been the development of interdisciplinary specialty teams with expertise in gender-affirming care. Clinical ethicists can play an important role on these teams in helping gender variant patients and gender-affirming providers navigate complex ethical issues, creating opportunities for enhancing patient experience, and easing provider moral uncertainty. Many opportunities exist for clinical ethicists to lend their skills to this area of clinical care. It is important for interdisciplinary transgender health care teams and other health care professionals providing transgender-specific care to understand the ethical issues involved in such care, the ways in which ethics expertise can be a resource, and the benefits and drawbacks of integrating a clinical ethicist into their team.

## Introduction

One of the distinctive trends in transgender health care has been the development of interdisciplinary specialty teams with expertise in gender-affirming care.^1–4^ One such program is our Transgender Surgery & Medicine Program at Cleveland Clinic. These programs create access to gender-affirming health care providers of different specialties within one institution and provide a gender-affirming space for patients who often face discrimination and stigmatization in the clinical setting. Our program has benefitted from expanding the scope of our interdisciplinary team to include an ethicist, as we have found that unique ethical issues can arise in the provision of transgender care. This article aims to highlight these issues and to discuss the role that an ethicist can play within an interdisciplinary team in helping gender diverse patients and gender-affirming providers navigate these complex issues.

In referring to the “ethicist” throughout this article, we specifically mean an individual who is a clinical ethicist. That is, a person who has specialized training and skills in clinical ethics consultation and who is accustomed to navigating clinical scenarios. It should be noted that academic bioethicists and clinical ethicists alike continue to make important contributions to advancing queer bioethics, challenging notions of optimal care for transpatients, and uncovering the biases health care professionals may bring to the care of LGBT people.^5–8^ We will begin by illuminating the ways in which transgender medicine interfaces with bioethical concepts by highlighting some of the ethical issues our gender center faces. We will then describe the composition and advantages of the interdisciplinary transgender care team and describe how patients and providers can benefit from the integration of trans-competent ethicists into such teams. Finally, we will describe the opportunities for consistent involvement by ethicists in these teams and present the benefits and risks associated with the embedded ethicist model as it applies to transgender health care.

## Bioethics in the Provision of Gender-Affirming Care

Our gender center has faced a number of ethical issues since its inception. One of the more common ethical issues our team faces is whether hormone therapy should be initiated for a patient with medical comorbidities that may be worsened by exogenous hormone therapy. An example would be a male-to-female patient with a history of venous thrombotic events (VTE) who wishes to be on estrogen to support the feminization process. Without hormone therapy, a patient may continue to experience an unacceptable level of gender dysphoria, which carries mental health burdens and risks (such as the worsening of an underlying mood disorder). However, this concern must be balanced against the medical risks associated with using hormones.^9^ It is ethically appropriate to discuss risks, benefits, and alternatives with patients to ensure adequate understanding and informed consent, including discussing whether any risks can be mitigated with adjunctive treatments (i.e., concurrent anticoagulation therapy, forms of estrogen that carry a lower incidence of VTE, tobacco cessation, and/or psychotherapy).^10–13^ In cases like these, an ethicist can support patients' decision-making processes and help clinicians delineate when an intervention's burdens so exceed the potential benefits that it would be ethically supportable to delay the intervention to optimize medical readiness (e.g., through utilization of an appropriate adjunctive treatment), or when the risks of nonintervention are so great that a harm-reduction approach to proceeding with hormone therapy is ethically warranted.

In the pediatric context, our program sometimes encounters parental disagreement related to a minor patient's plan of care. For example, a transgender teenager may seek hormone therapy and have one parent opposed to this intervention. Under a pediatric ethics framework, caregivers have a responsibility to promote the best interests of the child.^*^ In most cases, the best interests of a transgender teenager will be served by dual-parent support. Data suggest that transgender youth who are supported in their identities and who have access to gender-affirming care have better mental health outcomes.^14–16^ That often means that the first step in cases of parental disagreement is supporting families in exploring their discomforts and educating them regarding the benefits of gender-affirming care for transgender adolescents. However, there are times when dual-parent support is unachievable. An ethicist can help sort out what constitutes the best interests of the child in a particular case and when it may be ethically supportable to proceed with hormone therapy over a parent's objection (so long as the state's law permits one-parent consent). For example, a teenager's need for hormone therapy to mitigate gender dysphoria may be exceptionally acute or the opposing parent may have less moral standing to forestall a plan of care (i.e., if they are not involved in the child's life to a significant degree).^17^

We also face a number of more novel ethical issues. For example, should a surgeon decline to perform genital gender confirmation surgery on an adult patient when there are concerns that this might not be the patient's autonomous choice, with such concerns arising from the patient's relationship dynamic with her partner? An ethicist can help a care team distinguish in their minds the fundamental issue of coercion by a partner (which is ethically problematic) from possible value judgments by a team member about a patient's relationship choices or sexual preferences (which is not an ethically supportable reason to deny care). An ethicist in this clinical situation can also help frame for a care team what questions and avenues of discussion are appropriately directed at a concern around pressure from a partner versus inappropriate curiosity or stigmatization by team members, and can provide guidance regarding the optimal process for exploring this concern (i.e., discussion with both the patient and their partner, as well as discussion with the patient alone, optimally by a psychiatrist or psychologist on the team as well the ethicist to disclose and discuss the team's concern and ethicist's involvement). This process can strengthen care providers' confidence that they are providing care in line with a patient's authentic preferences and can help assure a patient (who may feel that the team is being overly intrusive or paternalistic) that their relationship choices are not being inappropriately judged, but that health care professionals do have a responsibility to ensure that a patient's decision is an autonomous one.

## Interdisciplinary Transgender Specialty Teams

The provision of gender-affirming health care through interdisciplinary specialty teams is endorsed by the World Professional Association for Transgender Health (WPATH^18^) and practiced at several gender centers in the United States.^1^ These teams often include nurses or social workers who coordinate care, family medicine physicians, internists, or advanced practice professionals who provide gender-affirming primary care and hormone therapy, psychiatrists, or other mental health professionals who support patients' mental health needs and assess readiness to transition, and surgeons who perform gender confirmation surgery, among others.^1,4^ Gender centers with pediatric teams can include pediatric endocrinologists, psychologists, and psychiatrists, as well as adolescent medicine providers.^3^

This interdisciplinary team model for transgender care has several benefits. First, it ensures a gender-affirming environment for patients by offering care through providers with expertise in gender identity issues. Such environments contrast with the experience of many transgender patients, who often experience stigma or discrimination in their interactions with health care providers and the medical system.^19^ Second, it promotes coordination and continuity of care within the interdisciplinary team.^18^ Patients can receive all of their care—from primary care to different aspects of gender-specific care—in one place with providers who know each other, work collaboratively, and effectively communicate to advance the care of the patients they have in common.^1^ Finally, it improves confidence in mental health professional referrals regarding a patient's readiness to medically or surgically transition.^18^ This confidence can prevent the kinds of delays that sometimes occur when providers receive referral letters from mental health professionals with whom they do not have experience or who do not have gender expertise. Patients may thus experience fewer barriers to care when a referring provider is a trusted member of the interdisciplinary team rather than an outside provider who is not well known to the team.

Interdisciplinary specialty teams may also benefit from the inclusion of ethicists. Ethicist involvement can enhance the ethical culture of a gender center, give providers more space, structure, and support to explore their responsibilities to their patients and the ethical challenges that trouble them, and support patients in complex decision-making processes. Like other providers, ethicists should be fully integrated into the fabric of the team to optimize the benefits of ethicist involvement and to promote coordination and continuity of care with respect to the ethical axis of care.

Importantly, lack of cultural competence by providers represents a significant barrier to adequate care for transgender people and has led to suboptimal ethics consultation outcomes.^20,21^ Trans-specific cultural competence, or trans-competence, involves having respect for transgender people, creating welcoming care environments, and being knowledgeable about the clinical aspects of transgender care.^22,23^ Any ethicist involved in an interdisciplinary transgender health care team should be gender-affirming, have background in gender diverse health care interventions and practices, and not carry anti-transgender bias.

There is no mechanism for measuring whether ethicists or other gender-affirming providers are adequately trans-competent, nor are there formal pathways for training ethicists to attain trans-competence. Individuals can seek learning opportunities through transgender health-related conferences and symposia, webinars or online training modules, and by shadowing gender-affirming providers.^24,25^ Ethicists should familiarize themselves with important publications in the field, such as the WPATH Standards of Care, and keep up with developments in the scholarly literature.^18,22^ Being trans-competent also means listening to the voices of the transgender community—for example, through nonscholarly publications, conversations with transgender people and social media, and the work of transgender advocacy groups. Ethicists should be familiar with queer and transgender critiques of the health care system and the role of health care professionals in perpetuating cisnormative biases. Unconscious bias by its very nature is not something that one is aware of, and explicit and implicit biases carry negative health care outcomes for patients against whom such biases are held.^26,27^ Ethicists with known anti-transgender bias are inappropriate members of gender-affirming care teams, and mitigating these biases is in itself important ethics work.^28^ Ethicists, especially cisgender ones, working within transgender health should be receptive to criticisms of their implicit biases.^29^

It is also important to note that even though transgender care can sometimes involve thorny ethical issues, this does not mean an ethical issue will always manifest in the care of a particular patient. Clinicians and ethicists should be careful to avoid imposing inappropriate barriers to access in the name of ethical prudence, or creating metaphorical “hoops” for patients to jump through (e.g., requiring patients new to a team who have been on hormones for years to seek a mental health assessment before continuation of hormone therapy).^30,31^ Indeed, the vast majority of patients in our program receive care without any ethical concerns whatsoever. Nevertheless, when ethical questions do arise, both gender-affirming providers and transgender patients may benefit from the normative expertise an ethicist who is trans-competent offers.

## The Ethicist's Role in an Interdisciplinary Transgender Health Care Team

The ethicist's role in an interdisciplinary transgender health care team can take several forms depending on a program's specific needs. For example, the ethicist can engage in formal clinical ethics consultation, providing recommendations to the team—often documented in the patient's chart—regarding the ethically supportable way(s) to proceed if an ethical challenge is encountered. As part of the consultation process, the ethicist will typically review the patient's medical record and speak with the stakeholders involved, which can include care providers, the patient, and sometimes their family (if appropriate and welcomed by the patient). In the preoperative case discussed above involving concerns that the patient's partner was coercing her into gender confirmation surgery, after exploring the patient's motivations for seeking surgery and how her relationship dynamic may (or may not) affect her desire for surgery, our team felt comfortable that her decision to seek surgery was an autonomous and self-directed one (that was supported by her partner). In this way, the ethicist's involvement helped us move the patient's care forward. One of the risks of involving an ethicist in the care plan is the patient perception that the ethicist is an additional gatekeeper to the patient attaining their therapeutic goals. To promote the therapeutic alliance between the patient and the care team, transparency with the patient regarding the ethicist's role and why they are involved in the patient's care is especially important to communicate.

The ethical issues that arise in the context of gender-affirming care do not always necessitate formal ethics consultation. Patient case conferences can provide the opportunity for the ethicist to apply an ethical lens to a patient's case and (re)orient the team's thinking by identifying the ethical considerations in play. This contribution represents a form of preventative ethics insofar as it may limit serious ethical dilemmas from developing down the road.^32^ It may also contribute to the general ethical culture of a program in a way that can guard against provider burnout by allowing providers to feel regularly supported from an ethics standpoint. Of note, there are times when an ethicist will recommend a course of action that a clinician disfavors or opposes; it is important to remember that supporting providers from an ethics standpoint does not entail always agreeing with them.

Ethicists can also contribute to the guideline or treatment protocol development process, especially when gender programs are in the early stages of shaping the contours of their practices. Our ethicist helped shape our adult and pediatric standard operating procedures by providing feedback and pushing back on some of the assumptions earlier versions contained. Teams may also wish to develop certain practices or standard processes for working through ethical issues that providers frequently face. Our ethicist supported an effort to establish our team's approach to caring for pediatric patients when parental disagreement exists. This process enabled providers with different perspectives to be heard, fostered discussion and understanding of the core values at stake, and produced a standardized process where *ad hoc* decisions were made previously.

Moreover, in some gender centers, the ethicist may be one of the few health care professionals who regularly interfaces with both the adult and pediatric teams. Pediatric and adult providers typically collaborate to achieve effective continuity of care when young adults transition from pediatric to adult teams, and ethicists who work with both teams can play an important role in bridging these programs and enhancing this process.

Finally, ethicists can bring a unique perspective to the development of research and scholarship on transgender health care issues. Not only can ethicists provide ethical guidance for thinking through research questions and study design^33^ but many ethicists who come from nonclinical backgrounds (e.g., philosophy, law, social work) can also contribute to the intellectual diversity of the conversation surrounding a specific research question. As our program considers what our future research efforts will look like, we plan for the ethicist on our team to collaborate to develop a research program that is ethically responsible and that asks questions that are useful for thinking about the ethical challenges presented in both the research context and clinical care.

## Risks and Benefits of the Embedded Ethicist Model in Transgender Care

The integration of ethicists into interdisciplinary transgender health care teams, like in other areas of embedded ethics, carries certain important benefits and risks when compared to a model that seeks ethics input on an as-needed basis from ethicists who are not integrated into the team.^34,35^ First, embedded ethicists who are trans-competent can contribute to the provision of culturally competent care and an affirming patient experience. As discussed above, ethicists who affirm patients' identities, have familiarity with the unique elements of transgender medical and surgical care, and are willing to reflect on their own potential cisnormative biases are best poised to be effective contributors to patient care.

Second, the inclusion of ethicists also empowers gender teams to proactively manage potential ethical issues.^35^ An ethicist may be part of the development of a standard process that helps prevent ethically problematic issues from manifesting, and providers can receive ethics input informally or during patient case conferences before a serious dilemma develops that requires a formal clinical ethics consultation. This means that ethical concerns can be addressed before they escalate into more serious ethical challenges, which makes for better patient care and alleviates burdens on both providers and patients.

Finally, this model enables ethicists to cultivate specialty expertise in transgender health care that translates into more effective ethical guidance. Ethicists who continue to acquire knowledge on best practices in transgender health care, who are familiar with issues important to the transgender community and trans-critiques of the health care system, and who are informed by their direct experiences with transgender patients will bring more expertise to their clinical practice, educational efforts, and gender-affirming scholarship. Trans-competent ethicists can teach other ethicists within their department and trainees engaged in ethics education about optimal gender care. They are also poised to train clinical ethicists in other health care systems in providing trans-competent ethics services.

However, there are potential risks to this embedded model. First, an ethicist may consciously or unconsciously attempt to align their perspective with those of other members of the transgender health care team to preserve relationships.^34,35^ This can occur if the ethicist is afraid of not being asked back to the team or receiving fewer requests for ethical guidance from the team if they too often disagree with the team's assumptions or opinions. In our program, this concern is partially mitigated by the ethicist reporting to leadership of the Center for Bioethics, which in turn reports to upper administration, rather than through individual medical or surgical centers or institutes, which allows the individual to have some level of independence.

Second, other health care professionals on the team may become overly dependent on the ethicist for minor ethical issues.^34^ Ideally, the process of clinical ethics consultation and the involvement of an ethicist in the clinical setting leads to the development of ethical competence on the part of other providers, who learn how to apply ethical principles and frameworks to similar ethically challenging situations.^35^ When an ethicist is “the person” on the team always contributing the ethical perspective, there is a risk that other providers may defer all ethical thinking to that individual. This risk can be mitigated by weaving educational components into the ethics consultation process, as well as by soliciting input from other team members regarding the values at stake or facilitating conversation around these issues, so that the ethicist's voice is not the only voice heard. While ethicists have specific expertise in ethical reasoning, their goals should include cultivating independent ethical thinking among the other team members.

Third, other health care professionals on the team may feel that their authority is being usurped by the ethicist. Under an embedded ethicist model, it is generally the team that invites the ethicist to collaborate or the relationship grows organically, so there is often some buy-in if not consensus regarding the benefits an ethicist brings to the team, as well as trust in that ethicist. Nevertheless, providers who join the team after the integration of an ethicist or who face a clinical situation in which the ethicist is recommending a course of action they disfavor or oppose may feel undermined. In these situations, it is important for the ethicist to remind providers that while ethicists apply their ethics expertise, it is always the provider's role to make medical judgments and that ethics recommendations (in most health care institutions) are advisory.

Finally, the embedded ethicist model may lead to providers only receiving a single perspective on the ethical issues that arise with their patients. Conversely, if a team were to reach out to its institution's ethics consultation service or committee, working with a different ethicist or group of ethics committee members each time, the team might receive ethical guidance that is more diverse.^36^ As such, one of the limitations of the embedded ethicist model is that the elucidation of the moral issues and recommendations for a particular ethical challenge may be less robust when coming from a single ethicist. However, a nonembedded ethicist may not have the same level of expertise and trans-competence when it comes to transgender care, and this is a trade-off to consider given the benefits that exist under the embedded ethicist model.

## Conclusion

In our gender program, we have found that the provision of gender-affirming care sometimes involves challenging ethical questions. Our interdisciplinary transgender health care team has benefitted from the integration of an ethicist in helping address these complex situations when they arise, in understanding the ways in which stakeholder values (for patients, clinician team members, other health care professionals, families, friends, health care institutions, the transcommunity, and society) present at a particular decision-point, and in designing care processes in ways that avoid perpetuating disparities and that mitigate the chances of more serious ethical issues developing. Gender-affirming providers may want to explore the ethics resources available to them and gender centers utilizing the interdisciplinary specialty team model may want to consider the ways in which the integration of an ethicist into their team can optimize patient care.

## Abbreviations Used

VTE: venous thrombotic events
WPATH: World Professional Association for Transgender Health

## Notes

^*^ Of note, there are different ways to conceptualize the best interest standard—as an obligation to ensure the best possible outcome for child, as a standard of reasonableness that achieves an adequate benefit to burden ratio, and as a requirement that disallows parental decisions that would seriously harm the child, among others. Kopelman LM. The best-interests standard as threshold, ideal, and standard of reasonableness. J Med Philos. 1997;22:271–289. Diekema DS. Parental refusals of medical treatment: the harm principle as threshold for state intervention. Theor Med Bioeth. 2004;25:243–264. McDougall RJ, Notini L. Overriding parents' medical decisions for their children: a systematic review of the normative literature. J Med Ethics. 2014;40:448–452. While debate continues regarding which framework is most appropriate under which circumstances, we consider a goal of maximizing benefits and minimizing burdens to be appropriate where a care team is deciding whether to initiate hormone therapy for a minor amid parental disagreement and with adolescent assent, and state intervention to override parental wishes is not under consideration. Shah SK, Rosenberg AR, Diekema DS. Charlie Gard and the limits of best interests. JAMA Pediatr. 2017;171:937–938.
